# Bodies of concern? A qualitative exploration of eating, moving and embodiment in young mothers

**DOI:** 10.1177/13634593211060760

**Published:** 2021-11-29

**Authors:** Grace Lucas, Ellinor K Olander, Debra Salmon

**Affiliations:** City, University of London, UK; City, University of London, UK; City, University of London, UK

**Keywords:** gender and health, maternity care, mental health, nutrition, research methodology

## Abstract

In some countries, including the United Kingdom, young mothers’ pregnant and postnatal bodies remain an area of concern for policy and practice, with interventions developed to support improved health behaviours including diet and physical activity. This article explores what young women themselves think and feel about eating and moving during and after pregnancy. Semi-structured interviews with 11 young mothers were conducted within two voluntary organisations. Data were analysed using thematic analysis with the theoretical lens of embodiment, which provided an understanding of how young women’s eating and moving habits related to how they felt about their bodies in the world. Four themes situated in different experiences of being and having a body were identified: pregnant body, emotional body, social body and surveilled body. Stress and low mood impacted eating habits as young women responded to complex circumstances and perceived judgement about their lives. Food choices were influenced by financial constraints and shaped by the spaces and places in which young women lived. Whilst young women were busy moving in their day-to-day lives, they rarely had the resources to take part in other physical activity. Holistic approaches that focus on how women feel about their lives and bodies and ask them where they need support are required from professionals. Interventions that address the structural influences on poor diet and inequalities in physical activity participation are necessary to underpin this. Approaches that over-focus on the achievement of individual health behaviours may fail to improve long-term health and risk reinforcing young women’s disadvantage.

## Introduction

Teenage pregnancy rates have declined in recent decades (Sedgh et al., 2015) but in many countries, including the United Kingdom, teenage pregnancy is still considered to be a public health issue (Coyne et al., 2013) with poor health and social outcomes reported for both mothers and their children (SmithBattle et al., 2017). However, this is contextualised by data that show that teenage conception rates are highest in the most deprived areas (Office for National Statistics, 2020) and that parents between the ages of 20 and 25, who tend to come from disadvantaged backgrounds, face similar negative outcomes (Action for Children, 2016).

In a neoliberal climate with emphasis placed on individual responsibility (Williams and Fullagar, 2019), interventions target improving health behaviours during and after pregnancy – as a time when women might be open to receiving health messages (Olander et al., 2018). Nutrition and diet have been one area of focus with young mothers (University of Southampton, 2017; Nielsen et al., 2006) as research suggests that they might not meet the particular dietary needs required to support their baby’s growth (Marvin-Dowle et al., 2016). Weight management interventions have targeted concerns around obesity before, during and after pregnancy (Haire-Joshu et al., 2015; Mei-Wei et al., 2017). Whilst there is limited research investigating levels of physical activity in young mothers, some studies suggest physical activity levels are low for young pregnant (Steinl et al., 2019) and postpartum women (Behrens et al., 2012) thus providing a further area of focus for health behaviour change.

Concerns about young women’s health behaviours have arguably led to an increasing emphasis on their pregnant and postpartum bodies as needing surveillance. Breheny and Stephens (2007) found that healthcare professionals view young mothers through a mix of ‘developmental’ adolescent and ‘good’ motherhood discourses, that are hard to fit together (p. 112). More broadly, a stigmatising framing of teenage mothers is dominant in some countries where becoming pregnant at a young age is antithetical to neoliberal self-management ideals (Cense and Ruard Ganzevoort, 2019).

As well as being at the centre of debate around the prevention of teenage pregnancy and health behaviour interventions to manage their bodies, young mothers are a part of broader socio-cultural narratives about women’s body image. As Nicolson et al. (2010) argue, although the pregnant body is a sign of the growing baby, health messages focussed on staying in control of weight during pregnancy and the pressure to get your ‘body back’ (p. 581) afterwards, reinforce the ideal body shape as thin.

Given the complex narratives attached to young mothers as bodies of concern, previous research has identified the importance of understanding how women wrestle with these representations (Barcelos and Gubrium, 2014). The current study aimed to further address this by exploring how young mothers feel about eating and moving in their bodies during and after pregnancy. Public involvement was sought via a group of young mothers drawn from the researchers’ university who were invited to a steering meeting to help develop the research design. In this meeting, young women shared their views on topics that could be discussed in the interviews, incentives for participation, images that could be used to stimulate discussion and some practical aspects of conducting focus groups and interviews. These interviews were conducted alongside a qualitative study of healthcare professionals’ perspectives on supporting young mothers with their eating and moving habits (Lucas et al., 2020).

## Theoretical framework

The bodily turn in social sciences and humanities criticism towards the end of the twentieth century foregrounded how bodies are constructed by their socio-historical contexts. This was followed by the material turn that sought to attend to corporeality and bodily feeling (Clever and Ruberg, 2014). The concept of embodiment usefully attends to these different perspectives as it moves beyond the idea of possessing the body as an object towards the sense of being in a body that is entangled with the world (Ellingson, 2019).

Embodiment was considered to be an appropriate theoretical lens for this study for a number of reasons. First, pregnancy is a unique experience where women may experience a different sense of their body-self (Warren and Brewis, 2004). Second, in adolescence, young women’s bodies are changing and teenage bodies are increasingly surveyed by society (Piran, 2017). Third, embodied subjectivity is resonant in the moving body. Previous research suggests that how a woman moves in her pregnant body brings to the surface changed proprioceptive feeling (Evans et al., 2016; Young, 1984). Fourth, eating habits can be driven by body sensations in hunger signals and in the sensory aspects of taste. Indeed, eating during pregnancy is about feeding both mother and baby, bringing with it ‘hormonal changes’ and ‘appetite increase’ (Taborelli et al., 2016: 319).

## Embodied researchers

Researchers working with embodiment have highlighted that researchers’ bodies are largely absent from their work (Brady, 2011) potentially further inscribing ‘their power over participants’ (Ellingson, 2006: 300). In our study with young mothers, who may have been subject to stigmatising teenage pregnancy discourse (Weed et al., 2015), we were particularly cognisant of this. However, at the same time as approaching the study as ‘embodied researchers’ (Sharma et al., 2009: 1642), we wanted to strike a balance in the research to retain a focus on the participants’ experiences (Chadwick, 2017). To achieve this, following Ellingson (2006), we provide short reflections on our own corporeal experiences considering how it felt to relate to the participants and how we experienced the spaces where we met and interviewed them. We also challenge Cartesian ‘mind-body separation’ (Ellingson, 2017: 5) by understanding bodies as fully entangled with selfhood.

## Methods

### Ethics

Ethical approval was received in March 2018 by City, University of London, School of Health Sciences ethics committee (ref. Staff/17-18/17). The study was conducted between May and June 2018 and involved semi-structured individual or paired interviews and focus groups with young women conducted in two voluntary organisations in England. We offered young women the opportunity to be interviewed individually or alongside other young mothers depending on what they preferred, as a way of addressing the reported imbalance of power in the researcher-adolescent relationship (Mack et al., 2009). Participants were informed that participation was voluntary, that they could withdraw their participation at any time up to the point of data analysis. Confidentiality was assured, unless relating to a risk of harm. All participants were given an identification number to ensure anonymity. No participants withdrew their data.

### Participants

Inclusion criteria for young women included being between age 16 and 25 with children under the age of 36 months, able to speak English and provide written consent. We included women up to the age of 25 as we felt that excluding women who were first pregnant over the age of 20 might omit valuable views, especially given evidence pointing to social inequality, rather than a particular age bracket, as a main determinant of poor outcomes (Action for Children, 2016). This view was also reinforced in our steering meeting with young women. After we contacted a number of organisations supporting young mothers, two voluntary organisations located through the policy and practice networks of one of the researchers agreed to be involved. One researcher (GL or DS) visited the organisations to provide information about the young mothers’ study, answer questions and leave contact details for further enquiries from potential participants. Key workers at the organisations liaised with young women, passed on the participant information sheets and a date was agreed for the researchers to attend and conduct the interviews.

### Interviews

Eleven mothers with children under the age of 36 months provided written consent to take part. Two women chose to be interviewed individually; the others took part in pairs or a group. All members of the research team (GL, EO, DS) were involved in interviewing, along with the additional support of a research assistant (AL). Where two team members were present, one conducted the interview and the other managed the visual prompts, as well as assisting the young women with their children. Interviews lasted between 30 and 47 minutes and were based around a topic guide, which covered questions about eating and moving during and after pregnancy. In total, we made four separate data collection visits to the sites. All the young women who took part were given a £15 shopping voucher for their time.

In keeping with our focus on conducting embodied research, we used three strategies. First, we took fieldnotes about our own interactions with participants and observations grounded in our sensory experiences. Second, we began the interviews with photo elicitation (Harper, 2002). The use of images during a research interview has been said to allow for more emotionally directed conversations (Rose, 2014) and arts methods have been previously used in research eliciting young mothers’ perspectives (Brady and Brown, 2013). In this study, images taken from UK public health materials – such as the ‘Eat Well guide’ (Public Health England, 2016), pregnancy literature and – given the surveillance of celebrity pregnant bodies (Nash, 2011) – images of pregnant celebrities were used to start the conversations. Third, with the aim of sharing control between participants and researchers (Ellingson, 2017), young women were also offered the option to bring their own images or to take photographs on their phones related to eating or moving in their daily lives. No young women in this study took up this option.

The interviews were not a disembodied process, but an interactive one between participants and the research team. As one of the researchers (GL) reflected in her fieldnotes:

As a researcher, I was aware of my body amongst participants’ bodies. As I entered the room, I felt a conscious shift in my demeanour – I wanted to convey that I was non-judgemental; that I understood. I felt that motherhood connected us. I could strongly empathise with the feeling of having a baby inside of my belly, or a wriggling toddler in my lap and the difficulty of trying to talk at the same time. But I had private space of my own to learn how to be a mother, whereas the room in the shared housing we entered was full of bodies – mothers, babies, children and staff, and in the summer heat it felt uncomfortably close. I was also aware of my body shape when talking about eating and body image how my ‘thin privilege’ might be perceived.

Young women’s bodies, children’s bodies and our bodies were all in the room together. Children do not sit quietly and babies do not observe without sound, so our conversations were frequently disrupted by noise and challenged by toddlers moving through spaces in which we were talking. Although this was difficult to negotiate as a researcher, it brought the reality of motherhood into the room, and as another researcher in the team (DS) reflected: ‘For these young women, issues connected to looking after themselves were not at the forefront as every waking moment was connected to putting the needs of the children first’.

### Analysis

Ellingson (2017), and others conducting embodiment research, describe the need to move away from positivist frameworks where bodies are considered a possible ‘contaminant of data’ (p. 37) and to take a flexible analytical approach. For this reason, thematic analysis (Braun and Clarke, 2006), which is not tied to a particular theoretical framework, was chosen. However, as Braun and Clarke (2006) emphasise, epistemological and other assumptions underlying the analysis should be made explicit. Although the approach to analysis was an inductive one, we paid close attention to sensory terms and descriptions of how participants’ bodies were felt and experienced. During the process, we also continued to discuss our experiences and reflections, acknowledging previous research that suggests the limitations of transcripts to fully translate the embodied interactions we had with participants (Ellingson, 2017).

Audio recordings of the interviews were checked against the transcripts. Interview data was coded using Nvivo 12 software. GL began the initial generation of codes and then identified and selected potential themes in the young women’s accounts. Fieldnotes were reviewed as the themes began to be identified, to see what these notes might contribute to the analysis. The collated extracts within each theme were then reviewed and checked – as suggested by Braun and Clarke (2006) – for ‘internal homogeneity’ (data within each theme was coherent) and ‘external heterogeneity’ (a clear distinction between themes) (Patton, 1990: 91). The data set was then reviewed again and the themes were reworked. After independent review by all members of the experienced research team (GL, EO, DS) and discussion and review, the themes were refined and renamed.

## Results

### Sample characteristics

Eleven young women participants were aged between 17 and 25 from Greater London and South West England. Participants were aged between 16 and 23 when they had their first child – with nine women becoming pregnant under the age of 20. One participant had three children, one had two children and all other participants had one child. Three participants identified as white and black Caribbean, one as black British, six as white British/white English and one as white Welsh. Ten participants were not in education or employment. All the women were currently or had previously been living in housing accommodation provided by the voluntary sector.

### Findings

Results are organised around four themes that are shaped by the ways that young women’s (YW) bodies are experienced in relation to eating and moving during and after pregnancy – pregnant body, emotional body, social body and surveilled body. These ways of being a body overlap and where relevant, the tension between these different experiences is described.

### Pregnant body – ‘whatever you fancy’

Pregnancy, for the young women we interviewed, came about during adolescence or young adulthood. At this time, young women’s bodies and food habits were experienced in the overlap of teenage desires along with a growing awareness of the needs of their developing babies. Young women, in our study, did not see pregnancy as a time to fundamentally change their diet. Whilst most young women cut out the foods they were told were unsafe to eat during pregnancy, three young women said they had not altered their diet at all. One young woman said she did not know about pregnancy food guidelines because she moved during her pregnancy and her care was affected and two others resisted the advice because they felt the information was always changing, ‘one minute, “it’s bad for you”, the next minute, “no that’s alright and this is bad for you”, so that’s why I didn’t really take it too seriously, I just carried on really’ (YW1). One young woman described how eating the food she liked was what mattered:

YW2: If I was told not to eat something, I still went and done it.

RES1: Oh, did you?

YW2: Yeah.

RES2: Was that because you didn’t believe them?

YW2: No, it was more about, probably about cheese, no, I’m not giving up that.

These decisions represented a moment when young women felt a tension between the freedom of what they wanted to eat as young people exploring their independence through food choices – eating ‘whatever you fancy’ (YW11) – and what they were told was best for their developing babies. Young women’s pregnant bodies were now also ‘surveilled bodies’ and some young women resisted this control, ‘I think I just ate what I wanted to eat, I still took my folic acid and stuff like that, like what I should take the vitamins, but I think I just ate what I wanted to’ (YW3).

Young women also discussed their different pregnancy cravings and expressed enjoyment at exploring them – they felt that their pregnant body was telling them what to eat (whether that was fruit or fast food):

YW5: Yeah you do get cravings for certain things, with my son I had cravings for KFC [fried chicken].

YW4: So yeah, when I was pregnant, I was really guilty of ordering takeaways, but I craved sweet food.

Cravings were largely described as positive sensations. A craving could be met with the food – something that could provide comfort during stressful circumstances.

In terms of doing physical activity in pregnancy, young women were led by how their bodies felt. Young women were aware of guidance about the benefit of physical activity but the pregnant body and its sensations (often connected to the ‘emotional body’ theme) dictated how young women acted on that guidance. In this time of inhabiting the pregnant body and before children were born, young women’s time was largely their own and their focus was mostly on what they felt they wanted to do. For some young women, this included going to the gym or swimming and continuing to enjoy physical activity. However, this was mediated by levels of tiredness as they experienced a shifting sense of their physical self. Participation in physical activity was also impeded by feelings of their growing bodies being judged by the world around them.

### Emotional body – ‘you get yourself into a loop’

Young women’s emotions were entangled with their eating habits and their sense of body-self. Food could constitute pleasure and young women, who took part in paired interviews and focus groups, enjoyed discussing their food preferences with others. However, the strongest expression of the emotional body was in terms of how low mood affected women’s feelings about themselves and how this influenced eating habits. Low mood was embodied in the belly as much as it circled around in the head:

When I get upset, I eat, when I’m upset, I eat and eat and eat and eat, until my belly hurts. And then, yeah. . . and then I’ll start crying because my belly hurts as well. So yeah, that’s just me (YW6).

Comfort eating provided temporary relief from stress but young women perceived that this added up to unwanted weight gain, which then worsened self-esteem:

You get yourself into a loop, you’ll sit here and eat rubbish and feel sorry for yourself, you’ll think you’ve put on some weight in the next day, so you stay in again, and eat some more (YW1).

After the birth of their babies, young women noticed a difference in their body shape and worries about weight were described by several participants, ‘I haven’t even got a set of scales cos I’d get upset’ (YW1). Whilst images of young celebrity mothers who appeared to just ‘bounce back’ compounded women’s low sense of self-worth (YW7), young women were more worried about how they were viewed by the people around them:

Because before they used to see me with like, not everything, but they used to see me when my body shape was so much different. And then now that. . . like after I’ve had the baby I feel like, ‘Oh, people are looking at me and talking about me’ (YW6).

Negative feelings about weight and body shape were caught up with a sense that there was social judgement of young motherhood, ‘I just feel like they are looking at me, “Oh look at the way your son’s dressed, look at the way you’re dressed”’ (YW10). Perceived judgement as a young mother also came from a feeling of being different to older mums, ‘I feel like they’re just looking down at you and, I went to the [parent and baby group] one and they just kept on giving me these looks’ (YW10). Young women’s understanding of how they were regarded by others stopped them socialising or taking part in organised activities – including physical activity.

Young women in our study, who experienced depression, explained it was hard to find the motivation to get out:

I kind of find myself in a downward spiral, I get down because I’m not doing anything and then I don’t want to do anything, because I’m feeling down and then it just gets me down (YW1).

As a result, young women felt increasingly socially isolated, ‘If you’re depressed and that, you just want to stay in your pyjamas, you lock yourself in and stuff like that’ (YW3). Depression also impacted eating habits as cooking and eating could feel like a chore and so they ate less, as one young woman explained:

Because I suffer with depression [. . .] it affects everything like, it affects my mood, how you eat, because sometimes I get so stressed, I got myself all worked up and I don’t want to eat really. Well, even if you don’t eat, you still have to feed your child, you know what I mean (YW7).

Once young women got out and moved, it helped their overall sense of wellbeing, ‘it’s like a domino effect, as soon as I got into doing some exercise, I got out and started getting a bit of a social life, it all kind of just sorted stuff out you know’ (YW1). However, even with strong personal motivation to move and feel better, structural situations (as reported in the ‘social body’ theme) often inhibited young women’s freedom to move.

### Social body – ‘doing it all by myself’

Drawing from embodiment theory, the social body theme illustrates how bodies relate to others and to the world around them and how habits and behaviours like eating and moving are shaped by social encounters, relationships and socio-economic structures.

Young women were focussed on caring for their children and wanted to cook healthy food and give their children a good start in life. However, living arrangements, finances and the lack of social support networks meant that they were often left to manage on their own, impeding their ability to do this. Young women described how they budgeted for food and planned meals but the cost of food was a dominating factor:

YW7: Right, now I need to go food shopping, so. . . I’ve already planned this week, because I’ve wrote in my notes, of everything that I’ve got, so what meals I have to get, what things I do have.

YW6: Junk food is more cheaper than healthy food.

YW7: Yeah, that’s true, that is true.

Most of the young women we interviewed were living in supported accommodation meaning that they cooked in shared kitchens and had little physical space to make and eat food. The locality where they were housed also had a direct impact on day-to-day food choices, ‘there’s not that many shops and stuff [. . .] it’s mainly just takeaways and Lidl’ (YW4). Young women’s ability to enact ‘healthy’ lifestyles was prohibited by the physical circumstances in which they bought food, cooked and ate.

Young women’s living circumstances often sprung from stressful events related to relationship breakdowns either during, or shortly after, the birth of their children. Several young women reported that they had little support from their children’s fathers, ‘nowadays, a lot of dads, you don’t really stay with the dad, if you do, you’re lucky, and if they stick around, you’re lucky as well’ (YW4). These stressful circumstances took a toll. For women who had tried breastfeeding, for example, a lack of emotional support had a direct impact on their bodies, ‘No, with me, I wanted to breastfeed, I breastfed for like a week, and then I gave up [. . .] I couldn't smoke and breastfeed, and because having a new born baby was very stressful, so yeah, I started smoking a lot’ (YW6). Stress felt through the ‘emotional body’ combined with the challenge of a lack of social support impacted this young woman’s ability to continue breastfeeding and her story was echoed by others.

Young women did not have wider support networks to allow them to get out and exercise, as one young woman explained, ‘I’m just stuck in all the time’ (YW1). After pregnancy, the (lack of) freedom to move highlighted how young women’s lives had changed, ‘I’ll go running for like an hour but who’s going to have [my toddler] for an hour while I go running off down the cycle path?’ (YW5). Young women described an intensity to their day to day existence, where their own needs were deprioritised. One young woman described how she fed herself at the very end of the day, ‘put something in the oven quickly. Eat it quickly [. . .] I’m doing it all by myself. I don’t have time to breathe’ (YW7). When women felt unsupported, their food choices were often based on what could be obtained quickly and cheaply. Food was often reported to be bought and cooked alone, mealtimes lacked social connection.

Despite some of these challenges, young women in our study were resourceful and determined and explained how they had learned to cook. Some had gained these skills from earlier family and community experiences but others had to teach themselves:

YW7: I love it, I cook everything from fresh, all my meals.

INT: And how did you know how to cook? Had someone taught you to cook?

YW7: I taught myself. I had to.

Young women showed their capacity to move through difficulty but showing resilience and doing it alone had its limits and self-management of their bodies was often the last thing on their list.

### Surveilled body – ‘we are here for the kid’

Health and social care professionals played an active – and varied – role in supporting young women’s eating and moving behaviours. Young women discussed the roles of their midwives (providing care during pregnancy, labour and early postpartum), health visitors (providing family support until children are five), family nurse practitioners (FNP) (specialised working with young parents through pregnancy until children are two) and social workers.

During pregnancy, young women explained that midwives had tried to help them to improve their eating and drinking behaviours. One young woman provided an example of this experience:

My midwife said to me at my, I think it was my eight week appointment, she said to me, ‘Oh how much coke do you drink?’, and I just looked at her and said, ‘A whole two litre bottle every day’, and she went, ‘I think you should cut that out’, and I did for the first few weeks and then I was just like, ‘I really can’t do it’. She said, ‘You can have a cup of tea, like just one, depending on how strong you have it’ and I was like, ‘Well, I literally didn’t have any milk and I have it really strong’. So, she was like, ‘Well, yeah you’re probably limited to like one’ (YW5).

Conversations like this, which were focussed on telling young women to cut down on sugar or caffeine, could feel like a power negotiation. For some young women, this experience as a ‘surveilled body’ made them feel more resistant to the advice, as they sought control over their own choices, especially because they felt led by powerful cravings from their ‘pregnant body’. After their babies’ birth, young women explained that health visitors were mostly interested in helping with food choices for their children, rather than how young women were eating:

They’ll ask the odd question, ‘Are you eating well?’ That’s all they’ll say. . .But then it’ll be more of the kid’s health, that’s definitely more what they’re focused on, and they will say, ‘We’re here for the kid’ (YW1).

Young women with toddlers commented that they had come to realise that they needed to think more about their own diets, as their children showed interest in what they were eating. All young women discussed how they wanted to provide nutritious food for their children – one young woman was keen to tell us that she cooked from scratch for her child and reiterated it a number of times. Young women perhaps felt they had to demonstrate they were a good mother, even within the context of a focus group.

For some women, support from several different professionals felt like interference, ‘I got too much support it’s driving me insane. I just got loads of people in my face and it’s like let me just be a mum, let me do what I need to do’ (YW10). Other young women had become wary of opening up to healthcare professionals as they felt they would be judged:

Yeah, there wasn’t really anyone to talk to or anything like that and you just didn’t want to say too much, because you didn’t want people to ‘Oh, she isn’t right with her kid’. I just didn’t want someone to think that, because if I didn’t have [child], I don’t know where I’d be. So, it’s kind of like, I think I blocked a lot in anyway, just so they’d think I was fine (YW3).

An exception to this was where young women had an FNP providing their care. Young women explained that FNPs put a specific emphasis on their wellbeing and their support was felt to be helpful and encouraging:

No, but she wants to make sure I’m getting the best care for myself she is actually really good and if I’m saying should I do this, she’ll give me the best advice she can. She said she can’t tell me what to do but she can give me the best advice of what I should do (YW10).

Generally, this kind of holistic support was seen as lacking by women in our study. Most of the young women reported that healthcare professionals were understandably stretched, appointments were often rushed and some services, such as cooking classes, had closed due to funding cuts.

## Discussion

In this study, through the lens of embodiment, we were able to understand young women’s food choices and moving habits as both somatically and socially influenced. Embodiment helps to dissolve the boundary of body and world as, people’s embodiment is ‘mediated’ by ‘continual interactions’ with others (Weiss, 1999: 5). For young mothers, inhabiting a role sometimes negatively perceived, judgements about pregnancy and motherhood accumulated and shaped how they felt about their bodies and lives affecting their sense of how to move and be in the world.

Young women, in our study, were not driven by cognitive rationalisation about long-term health benefits of eating or the nutritional aspects of food but by taste and satiation. This resonates with research that finds that, in the UK, teenage girls have the worst diets (Bates et al., 2017). Although young women were aware of culturally dominant moral discourse around healthy eating (Williams and Annandale, 2020) – and were largely cognisant of the need to adhere to the guidelines of what foods were safe to eat in pregnancy – at this time, diet choices were primarily steered by cravings, which young women used to guide their food choices. Previous research shows that pregnant women do not follow pregnancy advice without question but also attend to their own ‘physical embodied experience’ (Nicolson et al., 2010: 583). For some young women in our study, the physical sensation of craving foods in pregnancy appeared to provide them with an act of resistance against healthy eating guidelines. Taking control of food choices may be a particular focus for young pregnant mothers seeking autonomy during a time in their life when they feel they things are hard to control. Moreover, young women in our study also took ‘comfort’ from their favourite food choices, something that other research with teenage mothers has found (Strömmer et al., 2021). Whilst these feelings are not unique to young pregnant women, comfort and pleasure may have been important to young women in our study because of the challenging contexts of their lives. Desiring certain foods during pregnancy could elicit positive emotions, where women were aware of their bodies in a different way and enjoyed – as one young woman put it – ‘going with the cravings’. Young women were actively negotiating their appetites, which they understood as developing from their growing baby. In pregnancy, young women were experiencing their ‘insides as the space of another, yet [their] own body’ (Young, 1984: 30–31).

The ‘emotional body’ theme captured how young women’s eating and moving habits were linked to their mental health and wellbeing. For some young women, depression overtook intentions to cook and eat well. For other young women, comfort eating to address emotional stress felt like a circling ‘loop’ of unhealthy eating, low self-esteem and social isolation. Whilst young women did not discuss the word ‘stigma’ themselves, they were cognisant of being judged as young mothers and this had an impact on how they felt and moved in the world. Stigma is important in terms of eating and moving habits because, as Williams and Annandale (2020: 421) argue, it is ‘a reflexively embodied phenomenon’ that can negatively affect health outcomes. The relationship between the stigmatisation of young mothers’ bodies and behaviours that potentially worsen their health outcomes requires further exploration, particularly in light of neoliberal health ideals that push young motherhood into a category of ‘societal problems’ because of a, ‘failure to manifest the symbolic metaphors of neoliberal citizenship’ (Ayo, 2012: 104).

Embodiment scholarship has emphasised the entanglement of bodies with the world (Clever and Ruberg, 2014). Whilst the idea of the ‘social body’ has a range of meanings in relation to embodiment, in this study, the ‘social body’ theme identified how socio-economic factors as well as social support structures related to young women’s experiences of eating and moving behaviours during and after pregnancy. Most of the women we interviewed were living in temporary supported accommodation. The impact of housing instability on young parents’ stress levels and mental health has been previously reported (Smith and Roberts, 2011). In our study, living conditions were formative in how young women shopped, cooked and ate. Food environments in the local areas where women lived also shaped their food choices. Furthermore, financial constraints and a lack of childcare support restricted young women’s ability to engage in physical activity. Our research reveals the tension between the idea of the healthy ‘body project’ (Chrisler and Johnston-Robledo, 2018: 11) and the inequities faced by the young women in our study, in terms of access to the resources of, ‘time, money, physical effort’ (Ayo, 2012: 101). Structural and social factors were foundational in if and how they could adopt ‘healthy lives’ (Marmot et al., 2010; Williams and Fullagar, 2019). Our findings suggest that the social and structural underpinnings of poor diet and inequalities in participation in physical activity need to be addressed.

Without a strong social network, young women were arguably less able to cope with stress and self-esteem issues identified in the ‘emotional body’ theme. Young women were, on the whole, in regular contact with healthcare professionals throughout their pregnancy and postpartum. Where professionals had a holistic focus, young women expressed they were well-supported and valued their input. However, when diet and physical activity were discussed without a rounded view, young women felt judged about their choices and mistrustful of the information and guidance, ‘the do’s and don’ts’ (Ayo, 2012: 102) provided to encourage them to self-manage their health. The theme of the ‘surveilled body’ actively illustrated how a neoliberal approach to health behaviour can potentially have unintended negative consequences as young women either tried to assert control and challenge professionals or hide their problems. These findings suggest that broader support for young mothers should be driven by an embodied understanding of wellbeing with emphasis put on ameliorating social and structural issues (Lucas et al., 2019), rather than focussing on individual health behaviours in a targeted approach.

### Strengths and limitations

A key strength of this study was the way in which young mothers were given options for how they wanted to share their views – either individually on within groups – and the way photo elicitation was offered to participants. This builds on previous literature on the use of arts-based or participatory research with young mothers (Brady and Brown, 2013; Harlow, 2009) and the need to try to share power between researcher and participants. Given the complex narratives around teenage motherhood, a single interaction with young women was limiting and probably not going to undo their concerns about our possible judgement of their lives; repeat visits may have worked better to create a more trusting interaction. The young women we interviewed either were living (or had lived) in supported accommodation. These living circumstances influenced young women’s day to day eating and moving experiences and speaking to young women living alone, or with partners or family may provide different insights. Indeed, in order to understand the complex factors influencing food choices and eating habits, young women’s individual backgrounds and circumstances would need to be better understood. The use of fieldnotes was useful in putting our bodies back into the research process and strengthened our reflexivity, although how to represent these in the process of writing this article was a challenge and requires further reflection.

### Research and practice implications

Future research with young women might consider other dynamic embodied approaches such as digital storytelling (Barcelos and Gubrium, 2018) or body-mapping (Gubrium et al., 2016) to explore embodiment in more visceral terms. Furthermore, previous research with teenage mothers has reflected that because of stigmatising discourse around young motherhood, respondents might be defensive (Harlow, 2009). While we did not find this to be true, we noted that more than one young woman talked about how they wanted demonstrate positive parenting by taking part in the research, and were going to tell health or social care professionals about their participation. The influence of stigmatising discourses about teenage parenthood should be considered as researchers plan how best to engage with young parents’ views.

Indeed, in terms of implications for professionals working to support young mothers, this study has emphasised how the broader contexts of young women’s lives affect their eating and moving behaviours. Whilst healthcare professionals had reportedly advised young women about dietary choices through a behavioural lens, this had some limits, making young women defensive about being young mother or to feel like the complexity of their lives wasn’t understood. A holistic approach to support healthy eating and movement would start with young women’s bodies, and how they feel about them, and work to improve their sense of esteem, well-being and connectedness (which may or may not give the opportunity to share information about eating or moving). If young women are feeling better and less judged, their sense of being able to live actively and eat well may be increased.

As ‘bodies of concern’ young women may already feel criticised in terms of their status as young mothers. Approaches that over-focus on the achievement of individual health behaviours may fail to improve long term health and well-being and even risk reinforcing young women’s disadvantage.

## Conclusions

The study reported here explored young women’s views about eating and moving during and after pregnancy. Whilst the young women subscribed to notions of body management and control around eating, when they felt depressed, stressed or judged by a society stigmatising teenage pregnancy and bigger bodies, visceral feeling took over. Emotional eating and fears about weight gain could send young women into a spiral – affecting both their physical and mental health and wellbeing. In terms of moving, young women could not prioritise spending money on the gym and did not have childcare or social support in place to allow them to take part in formal exercise. Movement could help mental wellbeing, but young women’s complex lives made finding motivation and freedom to move difficult. How young mothers feel and experience their body in the world affects how they eat and move; support needs to begin there.
